# Experiences Among School Personnel and School Nurses on Educational Adaptations for Students With CFS/ME: A Qualitative Interview Study

**DOI:** 10.3389/fped.2021.756963

**Published:** 2021-11-11

**Authors:** Wenche Ann Similä, Torstein Baade Rø, Torunn Hatlen Nøst

**Affiliations:** ^1^Children's Clinic, St. Olavs Hospital, Trondheim University Hospital, Trondheim, Norway; ^2^Department of Clinical and Molecular Medicine, Norwegian University of Science and Technology, Trondheim, Norway; ^3^Department of Mental Health, Norwegian University of Science and Technology, Trondheim, Norway; ^4^Norwegian Advisory Unit on Complex Symptom Disorders, St. Olavs Hospital, Trondheim University Hospital, Trondheim, Norway

**Keywords:** chronic fatigue syndrome, education, social, school teachers, counselors, school nurse

## Abstract

**Introduction:** Chronic fatigue syndrome (CFS/ME) is a disabling disease severely impacting school attendance, education, and social life in young students. Uncertainties surrounding CFS/ME etiology may impact the interpretation of CFS/ME in schools. Thus, school personnel need information from health care providers to make adequate adaptations to education and social life at school for these students.

**Objectives:** To explore teachers, counselors, and school nurses' experiences with adapting education for students with CFS/ME aged 13–19 in secondary and high schools.

**Design:** A qualitative study with focus group interviews and individual interviews performed face-to-face or digitally between November 2020 and March 2021. Data were analyzed using Systematic text condensation.

**Participants:** Six teachers, two counselors, and four school nurses in secondary and high school participated.

**Results:** Adapting education for students with CFS/ME was challenging, especially before the students received a diagnosis. The challenges were related to identifying the students' adaptational needs, maintaining a teacher-student relationship due to school absence, difficulties in maintaining continuity of education, and uncertainty regarding the diagnosis. Successful adaptations were related to quickly reacting to school absence, early referral to educational, psychological services, a close collaboration with the school management, and the development of digital teaching for students with CFS/ME. Interdisciplinary collaboration and a clear, constructive plan with adaptive measures, including maintained teacher-student communication and educational and social adaptations, may be useful in preventing the losses, young people, with CFS/ME experience.

**Conclusion:** Early interdisciplinary collaboration to adapt education and social life at school for students with CFS/ME, may support teachers, counselors, and school nurses in their efforts to adapt education and prevent losses related to academic and social development in students with CFS/ME.

## Introduction

Young people's chronic health conditions may impact their health-related quality of life, academic performance, and social development (1–3). Identification of disease-related impairments with subsequent needs of educational adaptations is essential to ensure that these young people attend and continue schooling (4). Usually, the teacher is responsible for making the necessary adaptations to achieve this (5). Common adaptations include alternative tasks and home tasks as required, maintaining contact between teacher and student during school absence, and ensuring appropriate information exchange between teachers, parents, and health care providers. Management of these adaptations can be time-consuming for teachers, especially if they have several students with different chronic health conditions in their classes (6).

Chronic fatigue syndrome (CFS/ME) is a disabling disease with fatigue as the main symptom and a frequent worsening of symptoms after physical and cognitive activities, referred to as post-exertional malaise (PEM) (7, 8). Other symptoms are unrefreshing sleep, pain, cognitive impairments, and autonomic, neuroendocrine, or immune manifestations (7). The severity of CFS/ME symptoms differs in mild, moderate, severe, or very severe degrees, and may fluctuate along with the symptoms (8). The prevalence of CFS/ME is between 0.2 and 1.0% in young people (9, 10). Recently, it was found that post-COVID-19 might trigger CFS/ME in adolescents and young adults (11).

CFS/ME cause long-term school absence among young people (8, 12, 13), and for instance, school attendance fluctuates along with the severity of symptoms (14). Young people with CFS/ME suffer from physical, cognitive, and social impairments with severe impact on education and social life, and they need individual and flexible adaptations to participate in education and social life at school (1, 15–17). Adequate adaptations for students with CFS/ME are, i.e., later attendance, an opportunity to rest during the school day, fewer lessons, prolonged school progression, and facilitations to socialize with peers (1, 15). However, some students with CFS/ME are bedbound, socially isolated, and receive little help from their school (18).

Uncertainty surrounding CFS/ME etiology differs CFS/ME from other chronic health conditions (19). This uncertainty may impact the interpretation of the disease at school, and for this reason, the adaptations students with CFS/ME are offered may vary (20). Teachers need information and guidance from health care providers to successfully integrate students with CFS/ME in mainstream classes (4, 21). The school nurse may be an essential link between students, parents, teachers, general practitioners, and specialist health services regarding health needs for the students (22). Still, there is little knowledge about how school personnel and school nurses experience adapting education for students with CFS/ME.

Therefore, the aim of the study was to explore teachers, counselors, and school nurses' experiences with adaptations of education for students with CFS/ME aged 13–19 in secondary and high school.

## Methods

This was a qualitative study with a focus group and individual interviews conducted between November 2020 and March 2021. The study applied a phenomenological approach by focusing on the participants' lived experiences (23).

### Reflexivity

The authors were one nurse and one medical doctor experienced in assessing young people with CFS/ME in a hospital and a third researcher with experience as a nurse but no experience with young people with CFS/ME. All authors had experience with qualitative methods. Two pedagogues in secondary and high school gave input to the interview guide to limit the influence of the authors' preconceptions on the interview guide.

### Setting

In Norway, young people with CFS/ME under age 18 receive a diagnosis from specialist health care. Before CFS/ME diagnosis, the assessment includes thorough physiological, psychological, and social assessment and exclusion of other diagnoses. Generally, young people with CFS/ME are students in mainstream classes both before and after diagnosis. After diagnosis, school personnel and school nurses receive information about educational and social adaptations from health care providers. In the COVID-19 pandemic, the schools in Norway were locked down for physical attendance and offered digital education from March to June 2020.

### Informants and Recruitment

The sampling strategy aimed to include participants from geographically spread schools, including both rural and urban districts.

The study aimed to include at least 24–32 participants divided into four or five focus group interviews. As the COVID-19 pandemic made restrictions to meet in groups, and inclusion thus became challenging, it was decided to switch to individual interviews after two focus group interviews.

Invitations to participate in the study were sent by e-mail to 18 secondary schools, 15 high schools, and 12 school nurses in Mid-Norway. Inclusion criteria were being a teacher, counselor, or school nurse in secondary or high school and having previous or current contact with students with CFS/ME.

The first author contacted those who responded positively to participate with additional information about the study and to set a time for the interview. The study included four school nurses, six teachers, and two counselors from seven different schools in both rural and urban districts (Table 1).

**Table 1:** Characteristics of the informants.

**Characteristics**	***N*** **Tot (F/M)**
Occupation	
Teacher	5 (3/2)
Teacher with leadership responsibility	1 (1/0)
Counselor in school	1 (1/0)
Counselor in EPS^a^	2^b^ (2/0)
School nurse	4 (4/0)
Employment	
Employment in secondary school	9 (7/2)
Employment in high school	3 (3/0)
Participants experienced with 2 or more students with CFS/ME	9 (8/1)

*F, Female; M, Male*.

a *EPS, educational psychological counseling services*.

b *One teacher had previous experience from EPS*.

### Data Collection and Interview Guide

The first author, a female Ph.D.-student, interviewed all informants. One focus group interview with four participants was conducted face-to-face by the first author and one co-moderator present. The second focus group and individual interviews used a secure digital platform. All participants gave one interview. Notes were taken during and immediately after each interview. The interviews were conducted between November 2020 and March 2021.

The authors developed the interview guide for this study. The questions were based on previous literature (24), the experiences of young people with CFS/ME, and input from teachers experienced with young people with CFS/ME. The interview guide consisted of three main questions; “What, in your experience, is challenging for students with CFS/ME concerning the school?”, “What experiences do you have from adapting education for these students?”, and “How has the COVID-19 pandemic impacted the school day for students with CFS/ME?”. The open-ended questions allowed the participants to speak freely about themes related to questions not yet asked. The first and last author edited the interview guide before the individual interviews based on data from the focus group interviews. This edition supplemented the interview guide with questions on how the school personnel, school nurses, and other professionals cooperated when planning education for students with CFS/ME. Interviews were audio-recorded and transcribed verbatim. The transcripts included only the participants occupation. The focus group interviews lasted 75–86 min, whereas the individual interviews lasted 61–82 min.

### Data Analysis

The analyses were conducted with Malterud's Systematic Text Condensation (STC), a descriptive cross-case analysis strategy involving an iterative four-step analysis procedure developed from Giorgi's psychological phenomenological analysis (25). In step 1, reading all transcripts gave an overall impression of the data. In step 2, meaningful units in the transcripts were coded and grouped into five preliminary themes (Table 2). Step 3 included a systematic abstraction of the meaningful units by reducing the content into a condensate that maintained the informants' phrasings. In step 4, the first author wrote the analytical text and discussed it repeatedly with the co-authors, adding quotes to illustrate the findings. Table 2 provides an illustration of the analyses. Preliminary results were presented and commented on in a national interdisciplinary forum experienced with young people with CFS/ME and discussed with a research group on patient education and participation at the university consisting of researchers experienced with qualitative methods.

**Table 2:** Illustration of the four steps of systematic text condensation.

**Step 1** **Preliminary** **themes**	**Step 2** **Meaningful units coded** **and grouped in five** **preliminary themes**	**Step 3** **Condensed meaning** **(Preliminary themes were gathered in four main themes)**	**Step 4** **Final themes** **(Themes were renamed, and quotes were added)**
Distance relations expectation trust	**(1) Cognitive and emotional challenges in students with CFS/ME** “They fall outside while friends and peers move on,” “Students have lost confidence in school.” **(2) Relations with student and parents** “We communicate mostly with parents…,” “We do not know when the student will attend school,” “We are supposed to treat them as regular students”	**Lack of competence**We were supposed to treat the student as a regular student when he attended school, but this was often not possible because the student did not know what the class worked on. It has been challenging that the students were absent for an extended period and that we had little access to them.	**We experience that the students** **lose confidence in school**
Dilemma academically or socially Affiliation	**(3) Adaptations of education** “The assessment is year-long,” “Continuity of education is challenging when the student cannot meet regularly at school or are absent for such a long time,” “It has been a lot of trying and failing” (4) Social adaptations at school “The contact with school is important, they need to feel that they take part in things that happen”	**Demanding matters to be engaged in over time**The challenges lie in that the assessment period is often year-long. There are uncertainties around CFS/ME, and without knowledge, it has been difficult for us as teachers to make adequate adaptations for these students. It is necessary to focus on the social aspect as well as the academic. There has been a lot of trying and failing. Measures we have made often did not work.	**Adaptations of education before the student is diagnosed is challenging**
Helplessness Communication	**(5) Interdisciplinary collaboration.** “It is a relief when diagnosis is set, then we are informed from the hospital,” “Health care providers have a better awareness around CFS/ME now than previously,” “Information from health care providers is clear”	**Important with interdisciplinary collaboration.** It has been a great relief when the students are diagnosed, and we receive information from health care providers. We have experienced that health care providers have more knowledge about CFS/ME now than previously and that it is essential with a steady plan for adaptations of education and social life at school.	**Interdisciplinary collaboration is valuable but sometimes challenging**
Understanding	“Perhaps we should have started earlier,” “School absence is not problematized enough.”	School absence is poorly problematized. We do not need a diagnosis to initiate measures.	**Suggestions on successful adaptations**

The results were repeatedly checked against transcripts for validation. Identified themes and meaningful units were checked and recognized to verify that the final results were related to the original data. Microsoft Excel Version 2008 (26) and MindManager 2020 (27) were used as systematization tools during the analyses.

### Ethical Considerations

All procedures performed were in accordance with and approved by the standards of The Norwegian Center for Research Data (approval number 420197) and performed according to the Declaration of Helsinki. Consent was obtained from municipal directors to recruit participants among the associated municipalities' employees. All informants received oral and written information about the study and gave informed and written consent to participate. Each participant had a number used during the focus group interviews to avoid the use of names. Each participant was also de-identified by using of numbers in transcripts. The names of schools or districts were not mentioned in the transcripts. Data were stored and handled according to government regulations and the regulations of the organization responsible for the project.

## Results

In total, 12 participants, 10 females and 2 males, were interviewed (Table 1). Six of them knew their students before they got CFS/ME, whereas six had met the student only after they fell ill. The participants talked about experiences on educational adaptations for students from both before and after the students received the CFS/ME diagnosis. They had little experience with educational adaptations for students with CFS/ME from the lockdown of schools in the COVID-19 pandemic. The participants' experiences were analyzed and categorized into the four themes; *Adaptation of education before the student is diagnosed is challenging, We experience that students lose confidence in school, Interdisciplinary collaboration is valuable but sometimes challenging, and Suggestions on successful adaptations*.

### Adaptation of Education Before the Student Is Diagnosed Is Challenging

A perceived key challenge in educational adaptations for students with CFS/ME was the often year-long assessment process before the students received a diagnosis. This was a shared experience among all the participants. Teachers especially, spoke about educational adaptations. In this period, it was not necessarily straightforward for teachers to introduce educational adaptations because they lacked experience with CFS/ME and the necessary understanding of the student's needs. Therefore, they could easily make mistakes. However, if the teachers waited for a diagnosis before they initiated adaptive measures, students could be pressured to be more active than they could cope with and, thus, get worse. According to the school nurses, there were differences in whether teachers appropriately assessed the severity of the student's problems. Thus, early adaptations at school varied a lot. This could lead to unnecessary loss of education and social contact for the students, possibly followed by a lost sense of belonging to the school. In high school primarily, the challenges with adaptations were related to expectations of that students usually became more independent and made their own choices, while these students often became more dependent on others.

“(…) I can honestly say that the biggest challenge I experience as a school nurse with this patient group is before they receive the diagnosis (…) the period from a loss of function until they receive the diagnosis, it's so tough, the air flows out of the balloon.” (*School nurse III*)

Teachers found it challenging to maintain or establish a relationship with the students because of their student's high levels of school absence and hence, limited contact with the students. They usually communicated with the parents to reduce the burden on the student. Due to fluctuating symptoms, the teachers often did not know whether the student was coming to school or not on a particular day. When the student attended school, it was challenging to re-connect the student with the classmates because he often did not know what the class worked on.

“(…) It is difficult for me to keep track of what the student has learned academically, (…), did you attend the previous class, no you did not, you attended that one, but not the one before, so keeping track is very challenging, I think.” (*Teacher IV*)

### We Experience That the Students Lose Confidence in School

A shared experience among the participants was that they had met students who had lost confidence in school because their struggles were not taken seriously by the school and because expectations toward them were too high. For instance, one of the participants talked about a student who had to run 6 km during the physical education class because that was the scheduled activity that day. Another example was that missed training on key concepts could lead to embarrassment in the classroom when students had to answer questions requiring knowledge about the taught key concepts. During the classes, the informants had this in mind because the loss of confidence and embarrassments among peers could lead to school refusal in the students.

“It is like both unknown and scary waters (…) so if we (…) push too hard, have too large expectations that lead to a burden in the form of performance pressure, but at the same time does not have too low expectations so that he has nothing to reach for or something to be proud of (…). He cannot do everything. He must be re-engaged in some way, but it has to work. It is a somewhat delicate balance.” (*Teacher IV*)

The school nurses, especially talked about how their expertise in preventive health care could be helpful in this. By expressing needs on behalf of the students, they could contribute to a mutual understanding between students, parents, and teachers, thereby preventing the student's loss of confidence. In situations where teachers felt insecure, school nurses said they could provide support to reassure teachers about the adaptations they made for the students.

“It is not like we meet a lot of these students either, but for the individual teacher it will probably be the first time (…), so I think then we have more experience than them anyway (…).” (*School nurse IV*)

Even though the participants had experienced success with minor educational adaptations, the participants also experienced that the adaptations at school did not help much.

“(…) it becomes such a hopeless situation for a helper, we should not take over their problems, but there is great compassion for them, and that can be challenging (…). You have no prognosis or future, and it is so hurtful to deal with.” (*School nurse IV*)

### Interdisciplinary Collaboration Is Valuable but Sometimes Challenging

The participants experienced great relief when the students received a diagnosis. Subsequently, the students received follow-up from the hospital, and both teachers, counselors, and school nurses could dialogue with the hospital. For all participants, health care personnel had to communicate clearly to the school about the student's challenges. Participants, especially teachers with little experience, said they needed firsthand information to understand what adaptations could benefit students with CFS/ME.

“(…) I think the person who attends the collaborative meetings, like the teacher, will be the one who has the best understanding of the problem and the measures, (…) I feel I miss a bit because I do not attend these meetings with the medical staff and the EPS.” (*Teacher II*)

All participants highly valued the collaboration with health care providers because they were assured that the student's health was well taken care of, and they were guided on what measures to initiate. One participant with previous and current experience with CFS/ME, said that hospital personnel now referred to more evidence-based knowledge on CFS/ME regarding the need to focus on the social aspects and learning. Collaboration regarding students with substantial school absences was necessary for the participants. Nevertheless, interdisciplinary collaboration meetings could also be challenging.

“‘Between the devil and the deep blue sea’ can be the feeling because the school can be frustrated and the parents can be frustrated, and they are often frustrated with each other, so that enabling a good collaboration is sometimes challenging, because the parents have been coping with it for years, and the school thinks quite traditionally” (*EPS II*)

Another challenge regarding interdisciplinary collaboration, especially talked about by teachers and counselors, was related to different theories about what causes CFS/ME. In interdisciplinary meetings, they could spend a long time and use much energy discussing this issue. The interdisciplinary collaboration was nevertheless a joint project where the school had a significant responsibility to show interest and make contact, and where parents were the connection between the student, school, and health care.

“(…) It is so vital how the school copes with this situation when the student becomes so ill, and then I think specifically about the importance of a teacher making contact once a week, even if there is no teaching (…). Just the fact that he came by and said hello and talked a little (…) it meant so much to the student, (…) it was the lifeline to society in a way, and that he could send greetings back to the classmates.” (*School nurse III*)

The interdisciplinary meetings involved many professionals from both health care and educational systems. In addition, the attending professionals could change over time. The participants had experienced that the interdisciplinary meetings with many participants could lead to exhausted students and parents. Nevertheless, the interdisciplinary meetings were valuable, particularly if they agreed on a concrete plan with realistic educational adaptations.

“A concrete plan with specific goals (…) have their adapted plan where they in a way see what they are going to do, and to feel that they can attain the goal. Getting a confirmation that what they are doing is good enough and that it is what we expect (…) is important.” (*Teacher V*)

A summary of the challenges school personnel and school nurses had experienced is provided in Table 3.

**Table 3:** Summary of challenges related to adaptation of education and social life at school for young people with CFS/ME.

**Challenges before diagnosis**	**Students have lost confidence in school**	**Challenges in interdisciplinary collaboration**
Lacked experience and understanding of the student's needs.	Important to know how much you can push the student.	Frustrations such as obstacles to collaboration.
Lacked contact with the student.	Adaptations did not help much.	Uncertainty about CFS/ME etiology.
Students could be pressured to do more than they could cope with.	School nurses can support teachers with early adaptations.	Exhausting meetings.

### Suggestions on Successful Adaptations

The participants suggested ways for how successful adaptations could be carried out. For instance, counselors and school nurses pointed out that one measure could ensure that school absence was problematized early. Another suggestion was that the students were referred to Educational, Psychological Counseling Service (EPS) as early as possible. This presupposed that the school collaborated and trusted that EPS initiated the necessary measures in collaboration with the students and their parents. Teachers had experienced increased contact with students with CFS/ME by using digital teaching during the school lock down due to the COVID-19 pandemic. They suggested that this could be a continued measure beyond the pandemic if they had the resources to develop digital teaching for students with CFS/ME, for instance, as a tool for individual counseling time with the student.

“(…) measures we do for these students are to give work tasks that provide empowerment (…). Then I also feel that the student is motivated to do this again. (…) it may be to ask some leading questions or do something that they are a little familiar with, then, to make things safer (…) We have always placed the student with someone he knows well (…) I think it is a motivating factor to come to school as well.” (*Teacher I*).

**Figure 1:**
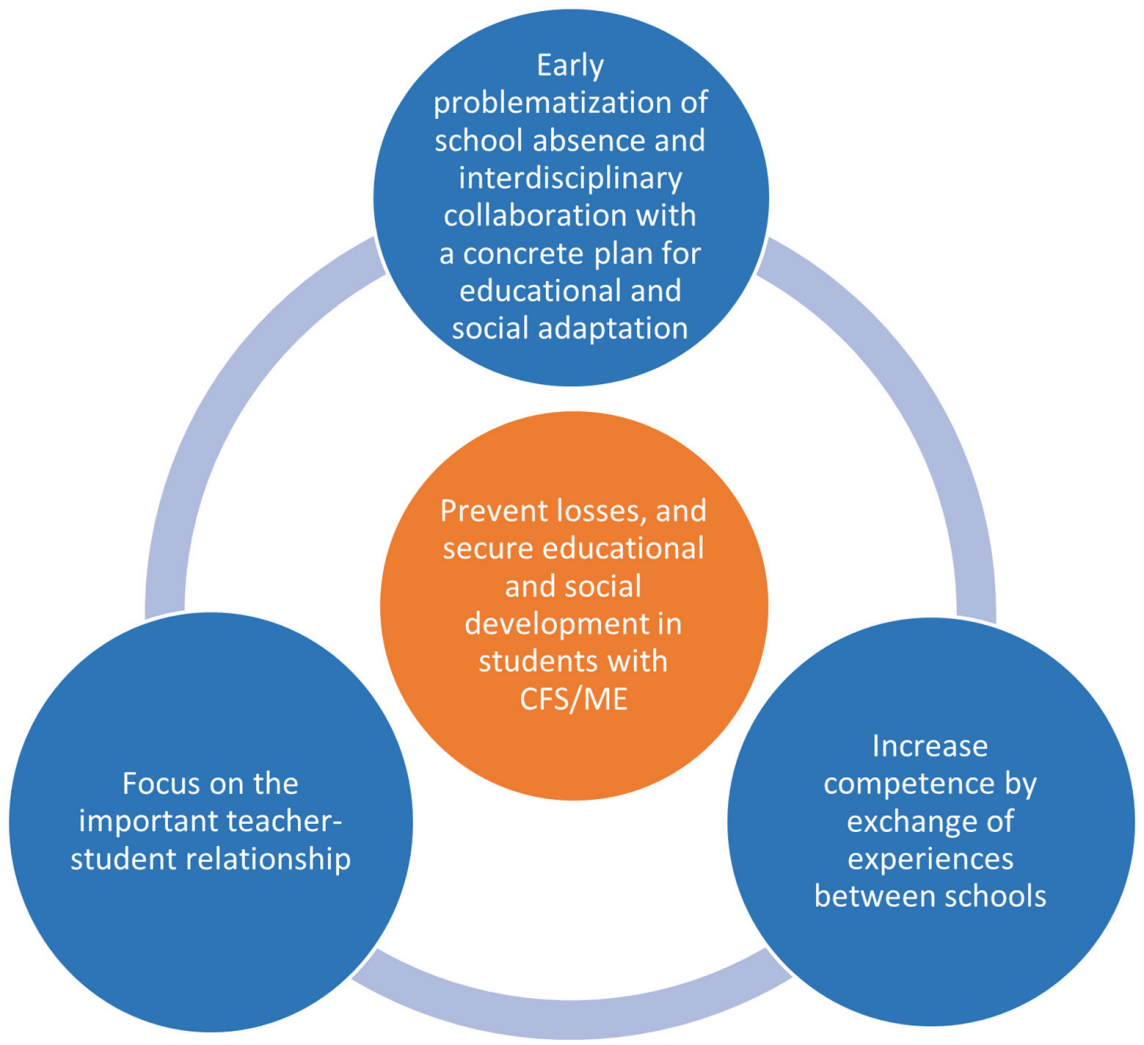
Suggestions for successful educational adaptations and prevention of losses for students with CFS/ME.

Because the participants, especially teachers, had only met a few students with CFS/ME, they needed more knowledge about suitable educational adaptations for these students. Related to this, they talked about the importance of collaboration between schools. One teacher said that now it seemed as if they were “reinventing the wheel” repeatedly in their respective schools. Through meeting students with CFS/ME, teachers gained experience and knowledge potentially beneficial for others. Therefore, they suggested it could be a good thing to have arenas where teachers could exchange experiences on educational adaptation for students with CFS/ME. They also said it could be helpful to meet students who had recovered from CFS/ME to hear their stories about what they considered to be beneficial adaptations at school. Figure 1 provides the participants' suggestions for successful adaptations.

## Discussion

In this study, we explored teachers, counselors, and school nurses' experiences with making educational adaptations for students with CFS/ME. The main findings were that they experienced challenges with educational adaptations, especially before a CFS/ME diagnosis. The challenges were related to school absence, few opportunities to meet with the students, and uncertainty about the diagnosis and the students' adaptational needs. This impacted the teacher-student relationship, challenged a maintained continuity of the student's education, and the student could lose confidence in school. Suggestions for successful adaptations were; early problematization of school absence and interdisciplinary collaboration on a concrete plan for adaptive measures; to focus on the important teacher-student relationship; and increasing competence in schools by exchanging experiences between schools. This could prevent unnecessary educational and social losses and secure a maintained development in students with CFS/ME. Recent experiences with digital teaching during the COVID-19 pandemic gave knowledge possibly useful for the development of adaptive measures.

### The Early Problematization of School Absence and Interdisciplinary Collaboration

Interdisciplinary collaboration was perceived as valuable when it provided necessary adaptive measures for students with CFS/ME. Continuity and well-defined plans for educational and social adaptations are of utmost importance for students with CFS/ME (1, 15). It also reassures teachers when they adapt education and relate to students with CFS/ME (1, 20). However, guidance on adaptive measures from specialized health care providers usually comes after a diagnosis is given (4). It has been highlighted that early recognition and diagnosis of CFS/ME and persistent long-term interdisciplinary follow-up are important to reduce morbidity in young people with CFS/ME (28). The participants in the current study suggested that early problematization of school absence followed by early adaptive measures could prevent losses of academic and social skills among students with CFS/ME. The current study confirms that interdisciplinary collaboration, including health care providers, is valuable for educational adaptations for students with CFS/ME. In addition, the study adds that early assistance from teachers and experienced health care providers may prevent some of the academic and social losses among students with CFS/ME.

Teachers, counselors, and school nurses experienced several challenges related to educational adaptations for students with CFS/ME, especially before they received guidance from specialized health care providers. Recently, it was found that teachers' efforts to introduce early measures had been based on the teacher's educational expertise, conversations with the students' parents, and intuition, rather than knowledge about adaptive measures for students with CFS/ME (20). This led to variations in the kind of support the students were offered. Another study found that students with CFS/ME experienced not being believed about their challenges due to a lack of knowledge about CFS/ME in schools (29, 30). Uncertainties regarding the CFS/ME diagnosis and etiology and a wide specter of symptoms that frequently overlap with other conditions have made it difficult for students with CFS/ME to explain their illness to teachers and peers (29). Without a proper understanding or adaptive measures at school, students with CFS/ME experience school absence, poor school-related quality of life, and reduced academic performance (31). The loss of education and social life may lead to depressive thoughts and anxiety in students with CFS/ME (29, 30, 32). Thus, teachers, counselors, and school nurses need education about how CFS/ME impacts academic performance and social life in affected students (1, 20, 33).

### Focus on the Essential Teacher-Student Relationship

Teachers experienced that it was challenging to maintain or initiate a teacher-student relationship, mostly due to the student's school absence. Thus, it was challenging to ensure continuity of the student's education. School attendance for students with CFS/ME may vary over time, and teachers often communicate with the parents instead of the student to minimize the pressure on the student (14, 20). It was previously found that students with CFS/ME who could not attend school struggled to be seen by their surroundings (34). Furthermore, increased contact with teachers was found to be associated with higher levels of health-related quality of life (HRQoL) in young people with CFS/ME (35). An important finding in this study is that teachers sometimes found it challenging to establish or maintain a relationship with students with CFS/ME, and that this may impact continuity of education. A closer teacher-student relationship might facilitate the teacher's understanding of the students' individual needs and ability to provide support (20). In addition, this may also be a possibility for bedbound students to contact the school society and receive help with education (18). Digital teaching may improve the teacher-student contact for students with CFS/ME and improve the understanding of social isolation among young people with CFS/ME (36). Therefore, it may be helpful to learn from recent experiences related to social isolation, digital teaching, and contact with students during the COVID-19 pandemic (37).

### Increase Competence in Schools by Exchange of Knowledge Between Schools

There was a limited exchange of knowledge between schools regarding adaptive measures for students with CFS/ME. A recent study describing the basis for teachers ' early adaptation of education for students with CFS/ME, did not mention the exchange of experiences between schools as a resource (20). Nevertheless, teacher collaboration and knowledge sharing can provide collegial support and improve educational adaptations for this group of students (38, 39), and this study supports that exchange between schools regarding successful adaptations of education for students with CFS/ME is a potential resource that is unexploited.

### Implications

Interdisciplinary collaboration around students with CFS/ME should be initiated earlier in the course of the disease, even before diagnosis. By problematizing school absences early, students with CFS/ME symptoms can be identified and receive adaptive measures to prevent unnecessary losses pertaining to their education. It is also important to focus on maintaining or establishing a relationship between teachers and students with CFS/ME. Digital contact may be a valuable resource for this. Exchange of experiences between schools about early adaptive measures for students with CFS/ME is a resource with potential benefits for teachers when they adapt education for students with CFS/ME.

### Strengths and Limitations

A strength of the study was the variation of experiences among the participants who represented three different professionality's in seven different schools. It was also a strength that the participants talked about both positive and negative experiences with the educational adaptations for students with CFS/ME. For reflexivity, preliminary results were discussed with others during the analyses.

A limitation to the study is that the sample was relatively small and limited to participants in Mid- Norway. If more districts and other countries were included, and the data collection period had been expanded, more participants could have been recruited, and more experiences could have been obtained.

The transfer from focus group to individual interviews made it possible to explore more expansive preliminary findings from the focus group interviews. Nevertheless, a limitation was that this reduced the possibility of obtaining data from the group dynamic in a focus group interview. Another limitation was that only a few participants represented high schools. Nevertheless, the findings include factors relevant both for secondary and high schools. A possible bias was that only participants with specific experiences registered. However, the various findings show that this was not the case.

It was a strength that a co-moderator participated in the first focus group interview, and a limitation that only one researcher conducted the digital interviews. However, the interviews were repeatedly discussed among the co-authors for input.

## Conclusion

Teachers, counselors, and school nurses found it challenging to adapt education for students with CFS/ME. If school absence is problematized early, and if teachers, counselors, and school nurses get early assistance from experienced health care professionals, it may be possible to identify students with CFS/ME symptoms and initiate early adaptive measures. Interdisciplinary focus on a clear and constructive common plan with adaptive measures including maintained teacher-student contact, and educational and social adaptations may prevent the losses among students with CFS/ME. Recent experiences with digital teaching during the COVID-19 pandemic may provide proper adaptive measures for use beyond the pandemic.

## Data Availability Statement

The raw data supporting the conclusions of this article will be made available by the authors, without undue reservation.

## Author Contributions

WS collected, analyzed, and interpreted the data and was the main author of the manuscript. TN supervised the data collection, analyses and interpretation of data, and contributed to the writing of the manuscript. TR supervised the project, analyses and interpretation of data, and contributed to the writing of the manuscript. All authors read and approved the final findings and the final manuscript.

## Funding

This work was supported by the Norwegian Ministry of Health and Care Services, St. Olavs Hospital, and the Norwegian University of Science and Technology.

## Conflict of Interest

The authors declare that the research was conducted in the absence of any commercial or financial relationships that could be construed as a potential conflict of interest.

## Publisher's Note

All claims expressed in this article are solely those of the authors and do not necessarily represent those of their affiliated organizations, or those of the publisher, the editors and the reviewers. Any product that may be evaluated in this article, or claim that may be made by its manufacturer, is not guaranteed or endorsed by the publisher.
